# The Salaries of Fever Nurses

**Published:** 1921-01-22

**Authors:** 


### The Salaries of Fever Nurses.

On page 398 we publish a scale of minimum salaries for the nursing staffs of fever hospitals which the Council of the Fever Nurses' Association recommends. In drawing up this scale the Council has been influenced by the following considerations, which it puts forward as the reasons for its conclusions:

(1) That the professional work of nurses has hitherto never been adequately remunerated, and the recommendations made are the minimum necessary for comfort in life and recognition of the work performed.

(2) That nurses holding qualifications similar to those held by sisters are, as health visitors, receiving from public health authorities from £175 to £300 per annum.

(3) That in view of the facts outlined in (2) it has been practically impossible for some time to obtain the services of women adequately qualified professionally and personally for posts on nursing staffs of fever hospitals. This is a matter of the gravest concern to those responsible for such institutions, and, in our opinion, can only be met by making the conditions more nearly approaching those pertaining in the ordinary outside world.

(4) That the staff run risks which they do not in other hospitals, and that they suffer a considerable degree of social ostracism from the nature of their work and from the fear of infection on the part of their friends and the general public.

A comparison of the minimum salaries recommended for fever nurses with those published last week recommended by the College of Nursing reveals some interesting points. In the case of probationers and junior assistant nurses in fever hospitals considerably higher salaries are recommended than those in the College scale, viz. £45 and £50 respectively. But even this is lower than the Metropolitan Asylums Board scale, which for probationers is £47 to £49, and for assistant nurses £47 to £66, with extra pay at special rates for any time worked in excess of a fifty-hour working week. The minimum salary recommended by the Fever Nurses' Association for sisters in fever hospitals is £80, rising by £10 to £120 (the same as the College scale), while night superintendents and home sisters in fever hospitals are to receive £100 to £150, the College minimum being £85 and £100 respectively in smaller hospitals, and £100 to £150 and £160 in hospitals with over 100 beds. Assistant matrons in the fever nurses scale are to receive £120 to £170, and in the College scale £85 to £255 according to the size of the hospital, whilst matrons in fever hospitals are to receive from £100 to £450 according to the hospital's size, the College scale being £150 to £500. It will be seen, therefore, that there is not any great difference shown between the two recommended scales, except in the case of probationers. The M.A.B. rate of pay for sisters is £77 to £83, for home sisters £91 to £96, assistant matrons £113 10s. to £141 10s., and for matrons £228 to £278, board, lodging, washing and uniform included for all except matrons, who do not receive uniform. Nurses in the M.A.B. service below the rank of second assistant matron work fifty hours a week, overtime being paid for at special rates.

